# Cross-species studies implicate the melanocortin 3 receptor more strongly in the control of pubertal development than energy balance

**DOI:** 10.1016/j.molmet.2025.102301

**Published:** 2025-12-10

**Authors:** Katie Duckett, Alyce McClellan, Laura J. Corbin, Irene Cimino, Ahmed Elhakeem, Ana Goncalves Soares, Alice Williamson, Eloise Cross, Zammy Fairhurst-Hunter, Slavé Petrovski, Debra Rimmington, Jesús Alegre-Díaz, Jaime Berumen, Pablo Kuri-Morales, Roberto Tapia-Conyer, BELIEVE study, Jacek Mokrosinski, I. Sadaf Farooqi, Asif Rasheed, Danish Saleheen, Adam S. Butterworth, Nicholas J. Timpson, Anthony P. Coll, Eleanor Raffan, Brian Y.H. Lam, Stephen O’Rahilly

**Affiliations:** 1 Medical Research Council Metabolic Diseases Unit and NIHR Cambridge Biomedical Research Centre, Institute of Metabolic Science-Metabolic Research Laboratories, University of Cambridge, Cambridge, UK; 2 Department of Physiology, Development and Neuroscience, University of Cambridge, Cambridge, UK; 3 MRC Integrative Epidemiology Unit at the University of Bristol, Bristol, UK; 4 Population Health Sciences, Bristol Medical School, University of Bristol, Bristol, UK; 5 Centre for Genomics Research, Discovery Sciences, BioPharmaceuticals R&D, AstraZeneca, Cambridge, UK; 6 Experimental Medicine Unit from the Faculty of Medicine, National Autonomous University of Mexico, Mexico City, Mexico; 7 Instituto Tecnológico y de Estudios Superiores de Monterrey, Tecnológico, Monterrey, Nuevo León, Mexico; 8 Center for Non-Communicable Diseases, Karachi, Pakistan; 9 Columbia University Irving Medical Center, New York, NY, USA; 10 British Heart Foundation Cardiovascular Epidemiology Unit, Department of Public Health and Primary Care, University of Cambridge, Cambridge, UK; 11 Victor Phillip Dahdaleh Heart and Lung Research Institute, University of Cambridge, Cambridge, UK; 12 British Heart Foundation Centre of Research Excellence, University of Cambridge, Cambridge, UK; 13 National Institute for Health and Care Research Blood and Transplant Research Unit in Donor Health and Behaviour, University of Cambridge, Cambridge, UK; 14 Health Data Research UK Cambridge, Wellcome Genome Campus and University of Cambridge, Cambridge, UK

**Keywords:** ALSPAC, MC3R, Puberty, Obesity, Melanocortins

## Abstract

Hypothalamic neurons expressing either POMC or AGRP sense nutritional state directly and indirectly and transmit these neuropeptide signals to other brain centres through the melanocortin 3 and 4 receptors. MC4R is primarily concerned with the control of appetite and energy expenditure while MC3R is more closely related to the control of linear growth and the timing of puberty. The role of MC3R in the long-term control of energy balance and body composition is less clear, particularly in humans. We have undertaken studies in humans, domestic dogs and mice with the goal of clarifying the relative impact of MC3R deficiency on energy balance, growth and sexual development. By studying three large consanguineously enriched cohorts, totalling approximately 300K people, we identified nine individuals who are homozygous for functionally null *MC3R* variants. The body mass index (BMI) of the homozygous *MC3R* variant carriers was not significantly different from that of age, sex and demographically matched controls, with six of the nine homozygotes having a BMI <30 kg/m^2^.

We detected a canine *MC3R* missense variant (p.M320I) which is common in labrador retrievers and showed that this significantly impairs receptor signalling. Dogs homozygous for p.M320I were lighter and showed delayed pubertal development but were not significantly more obese than wild-type or heterozygous dogs. We also established that the lack of *Mc3r* delayed pubertal development in both male and female mice.

Finally, we studied growth and pubertal trajectories of individuals carrying rare loss-of-function *MC3R* variants and found that male carriers had delayed peak weight velocity and genital development but had no evidence for excess body fat compared to non-carriers.

Our results support MC3R having a conserved role across mammals in controlling growth and pubertal timing. While MC3R deficiency may influence linear growth and body composition, complete loss of MC3R does not result in a penetrant human obesity syndrome.

## 1 Introduction

Two of the five melanocortin receptors (MCRs) are highly expressed in the brain, where they respond to both the agonistic melanocortin peptides derived from pro-opiomelanocortin (POMC) and the inverse agonist agouti-related peptide (AGRP). The production and release of these two classes of peptides are reciprocally regulated by signals conveying nutritional status. *MC4R* null mice and humans are obese, hyperphagic, and have lower energy expenditure, reflecting a key role for this receptor in the control of food intake and energy expenditure [1–3]. In contrast, mice lacking MC3R are not hyperphagic and do not develop severe obesity but have reduced linear growth and an increased ratio of fat to lean mass [4–6]. There have been many genetic studies attempting to understand the role of *MC3R* variants in human obesity (reviewed in [7]). In 2019, a meta-analysis of published studies suggested that heterozygous loss-of-function (LoF) mutations in *MC3R* were not likely to be the cause of a penetrant form of dominantly inherited obesity but individuals with obesity were more likely to carry a LoF variant in *MC3R* [8]. In 2021, we did not find any evidence of association between Body Mass Index (BMI) and approximately 2400 carriers of proven MC3R LoF mutations (p.F45S and p.R220S) in the UK Biobank [9]. Characteristic phenotypes found in those carriers included reduced height, lower scores on some indices of lean mass, and a delayed onset of puberty. In parallel studies in mice, we reported that MC3R was necessary for the communication of caloric state to the reproductive axis and that male mice lacking MC3R had a modest delay in reproductive maturity [9]. In the same publication, we reported a single individual homozygous for a missense mutation (p.G240W) which completely abolished the receptor’s signalling capacity. Consistent with what we had found in heterozygous individuals, that participant had markedly delayed puberty and short stature. He also had severe obesity (BMI = 40.4 kg/m^2^) and a body fat percentage of 48.5% [9].

Herein, we describe further studies undertaken to more securely establish genotype- phenotype relationships with respect to *MC3R*. In three human populations enriched for consanguinity, we identify participants who are homozygous for nonsense or functionally impaired missense mutations in *MC3R* and compared anthropometric phenotypes in homozygotes vs wild-type (WT) participants from the same population. Our previous work in UK Biobank relied on pubertal phenotypes recalled in middle age. Herein, we report the directly measured anthropometric and pubertal phenotypes of *MC3R* mutation carriers from a prospective UK based birth cohort.

Taking a cross-species approach, we also report the finding of a common, functionally significant *MC3R* missense variant in dogs and characterise the phenotype of canine carriers. Finally, we have extended our previously reported study of sexual development in mice lacking MC3R to allow us to make more robust statements about the impact of its deficiency on reproductive development of both male and female mice.

## 2 Results

### 2.1 No excess of obesity in humans lacking functional MC3R

To determine if homozygous carriage of a loss-of-function (LoF) mutation in *MC3R* causes monogenic obesity in humans, we searched for further individuals homozygous for potential LoF mutations in three exome cohorts enriched for consanguinity.

In the Pakistan Genomics Resource (PGR) of ~80k exomes, we obtained all *MC3R* coding and non-synonymous variants with a minor allele frequency (MAF) < 0.01 for which there were homozygous carriers: p.V63M, p.D84N, p.N91S, p.M97I and p.V144L (Table 1; Supplementary Table 1). In the BangladEsh Longitudinal Investigation of Emerging Vascular and nonvascular Events (BELIEVE) cohort, which has exome data available for ~72k participants, we similarly identified two variants present in 3 homozygous carriers: p.V63M and p.M97I. In a third population cohort of participants from the Mexico City Prospective Study (MCPS) cohort, which has exome data available for ~140k participants, a protein truncating variant (PTV) p.Y143* was found in 4 homozygous individuals.

**Figure 1:**
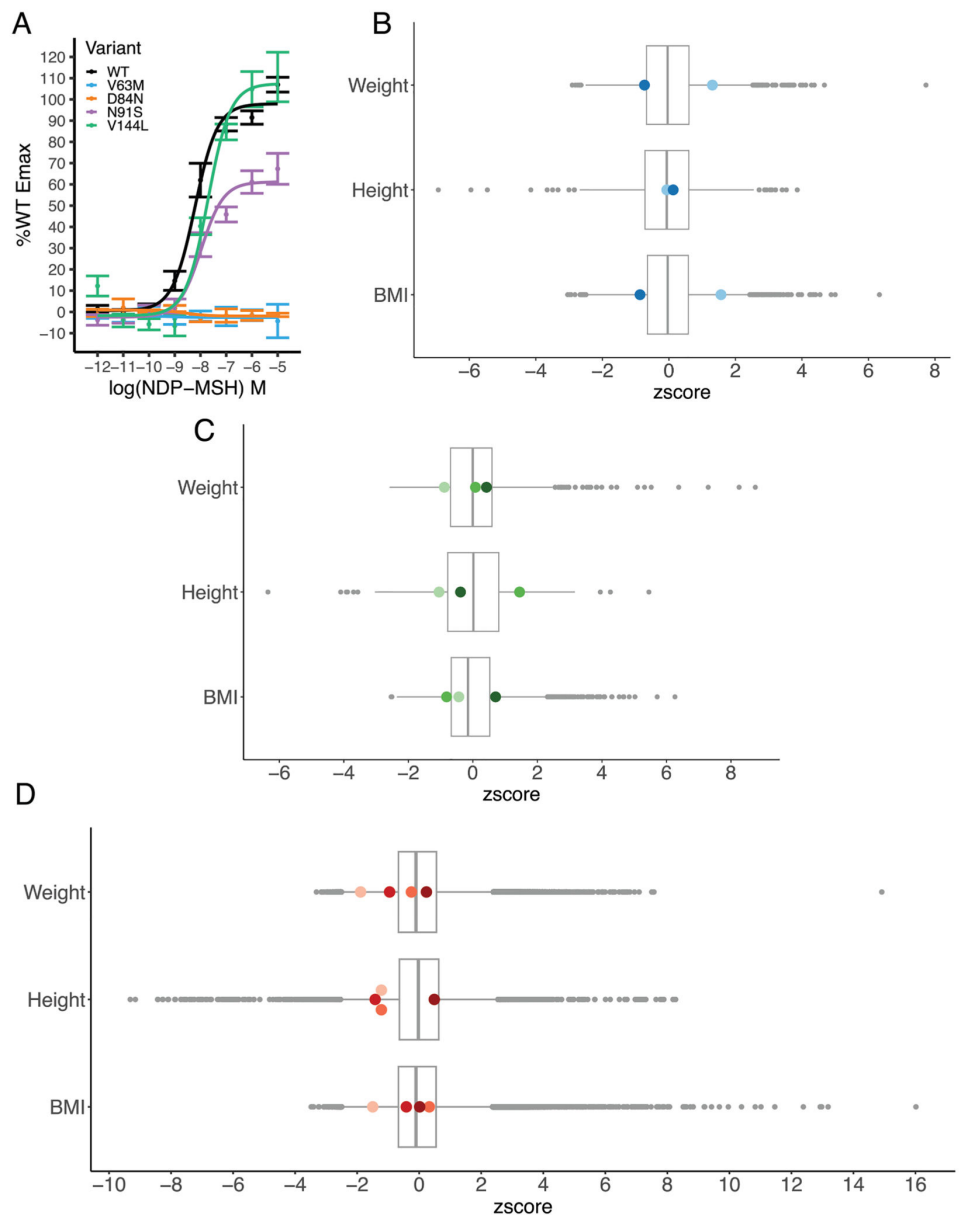
Characterisation of *MC3R* LoF variants and the weight, height and BMI of homozygous carriers. (A) *In vitro* measurement of cAMP response of overexpressed *MC3R* WT and homozygous mutations, upon stimulation with NDP-MSH ligand, reported as percentage of Emax of WT. Error bars = SEM. *N* = 3–6 biological replicates. (B—D) Z-scored plots of weight, height and BMI for the cohorts B) BELIEVE (two p.V63M homozygotes, in blue), C) PGR (two p.V63M and one p.D84N homozygotes, in green), and D) MCPS (four p.Y143* homozygotes, in red). Homozygotes for CLoF *MC3R* mutations are Z-scored against an age, sex and ethnicity-matched population, then Z-scores combined for plotting. Each homozygote is represented as a coloured dot. Boxplot represents median with 25th and 75th percentiles, and whiskers extend to 1.5 times the interquartile range. Outliers are plotted as individual greyscale dots.

M97I has been previously characterized to have WT-like cAMP activity [9,10]. The premature stop mutation p.Y143* is presumed to have no signalling activity as it would lead to deletion of the last four transmembrane helices. The remaining missense variants were characterized in this study by assessing cAMP activity in transfected cells (Figure 1A; Supplementary Table 2). Both p.V63M and p.D84N abolished cAMP production in response to the potent synthetic α-MSH analog, NDP-MSH. Therefore, 9 individuals (5 female, 4 male) across the three cohorts were homozygous for complete LoF (CLoF) mutations in *MC3R*.

We obtained data regarding their height, weight and BMI. When compared with age, sex and ancestry-matched wild-type members of their respective populations, none of these measures differed significantly (Figure 1B—D). Only 3 out of 9 individuals (2 female, 1 male) had a BMI greater than 30 kg/m^2^, with the maximum BMI being 31.7 kg/m^2^. No data on puberty timing was available in these individuals, although three women were reported to have children during their twenties.

Additionally, we did not find any homozygous *MC3R* variants in 1748 children (9% from consanguineous families) with severe obesity recruited to the Genetics of Obesity Study (GOOS), a cohort in whom homozygous variants in all obesity syndrome genes have previously been identified [11,12].

### 2.2 Evidence for delayed pubertal development but normal fat mass in carriers of LoF mutations in *MC3R*

**Figure 2:**
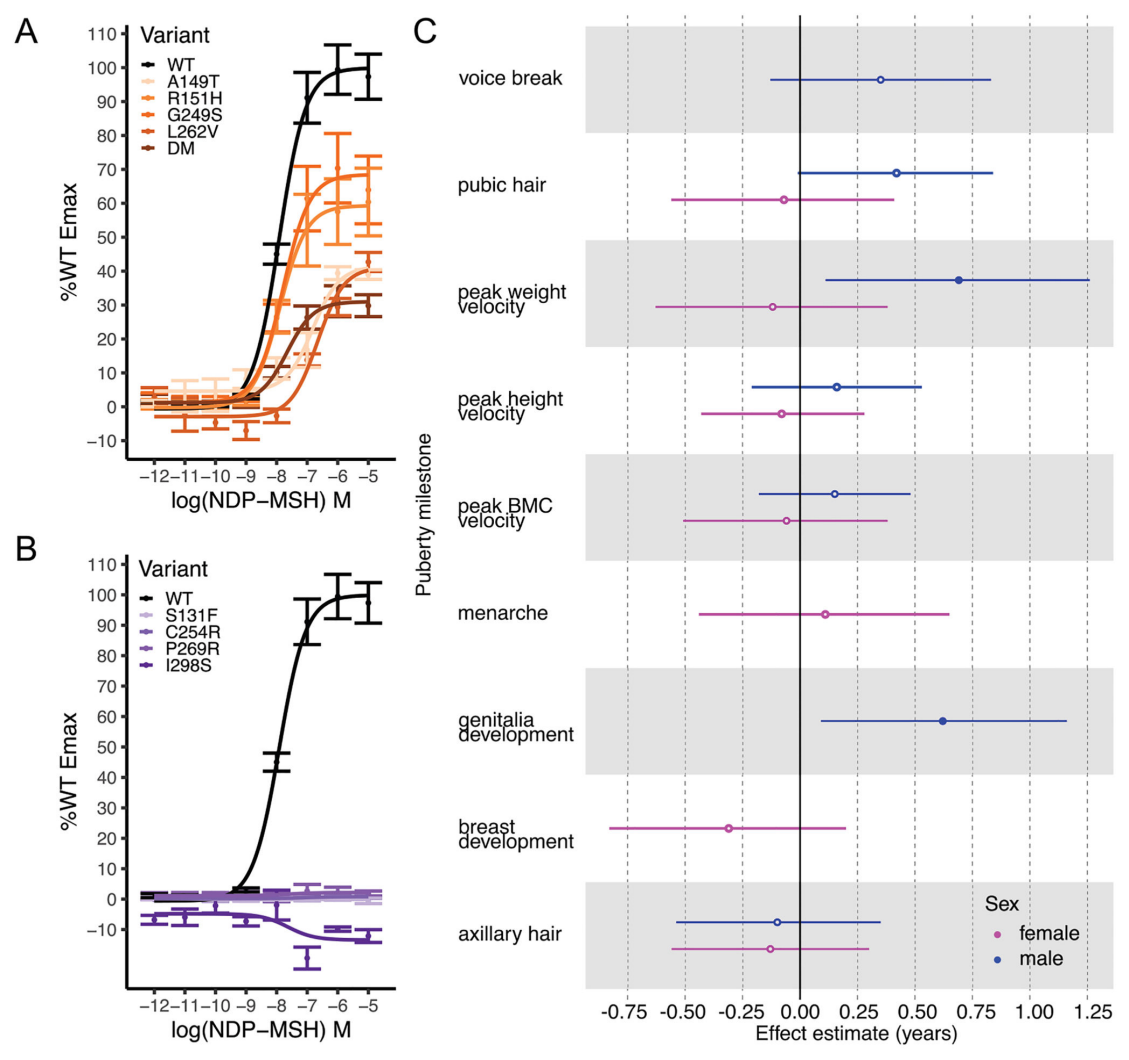
Loss of function *MC3R* variants in ALSPAC and carrier associations with pubertal milestones. (A—B) cAMP response of *MC3R* WT and heterozygous variants upon stimulation with NDP-MSH ligand, reported as percentage of Emax of WT, where (A) are partial LoF variants, and (B) are complete LoF variants. Error bars = SEM. *N* = 2–7 biological replicates. (C) For carriers of mutations in (A) or (B), results of linear models for 9 derived puberty phenotypes for females and males. Filled in circles represent unadjusted *p* < 0.05. Hollow circles represent unadjusted p ≥ 0.05. Lines represent lower and upper 95% confidence intervals. Numbers for carriers and non-carriers included in the analysis can be found in Supplementary Table 4.

To further elucidate how LoF *MC3R* variants impact puberty trajectories in children, we investigated the Avon Longitudinal Study of Parents and Children (ALSPAC) cohort, which has imputed genotyping data, whole exome sequencing (WES) data and longitudinal anthropometric and pubertal timing data across approximately 12,000 children [13,14]. We searched for coding variants (MAF<0.01) across the WES data and found 12 heterozygous (zero homozygous) variants, four of which were already characterised as LoF, and 8 novel predicted LoF based on combined annotated dependent depletion (CADD) score (Supplementary Table 3). We characterised these new missense variants *in vitro* to confirm their predicted effects, finding 4 exhibited partial activity (between 25% and 75% WT Emax activity) and 4 exhibited complete LoF i.e. no response to cAMP (Figure 2A−B; Supplementary Table 2). We found that 2 out of 3 carriers of p.G249S also carried p.R220S; this is consistent with GnomAD v2.1.1 where 15/16 p.G249S carriers also carry p.R220S. Therefore, we introduced both mutations in *MC3R in vitro* as a double mutant (DM) and found an approximately additive effect of these mutations on impacting cAMP activity (p.R220S Emax = 67%; p.G249S Emax = 71.0%; DM Emax = 30.8%) (Figure 2A). Based on the functional characterisation, a total of 58 individuals carried a *MC3R* variant which leads to either partial or complete LoF.

Puberty phenotypes were recorded in children and adolescents via questionnaire and have been derived into nine pubertal timing phenotypes [15]. Of the 58 carriers, 42 (20F, 22M) had recorded puberty data; we performed linear regression to assess if carriers had a difference in timing of any of the 9 puberty milestones compared to non-carriers (Figure 2C; Supplementary Table 4). Males showed a delayed age at peak weight velocity (beta = +0.69yrs, *p* = 0.02; male population mean ± SD = 13.6 ± 1.2yrs) and a delayed age at reaching Tanner stage 3 genitalia development (beta = +0.62yrs, *p* = 0.02; male population mean ± SD = 12.7 ± 1.2yrs).

We also assessed changes in height, weight, BMI, fat mass and lean mass from childhood to early adulthood for carriers vs non-carriers (Supplementary Fig. 1; Supplementary Table 6). A trend toward lower weight from adolescence to adulthood was observed, however the 95% confidence intervals overlapped with non-carriers for all anthropometric traits.

### 2.3 Dogs carrying a common LoF mutation in *MC3R* are lighter, have delayed pubertal development but no increase in obesity risk or fat mass

**Figure 3:**
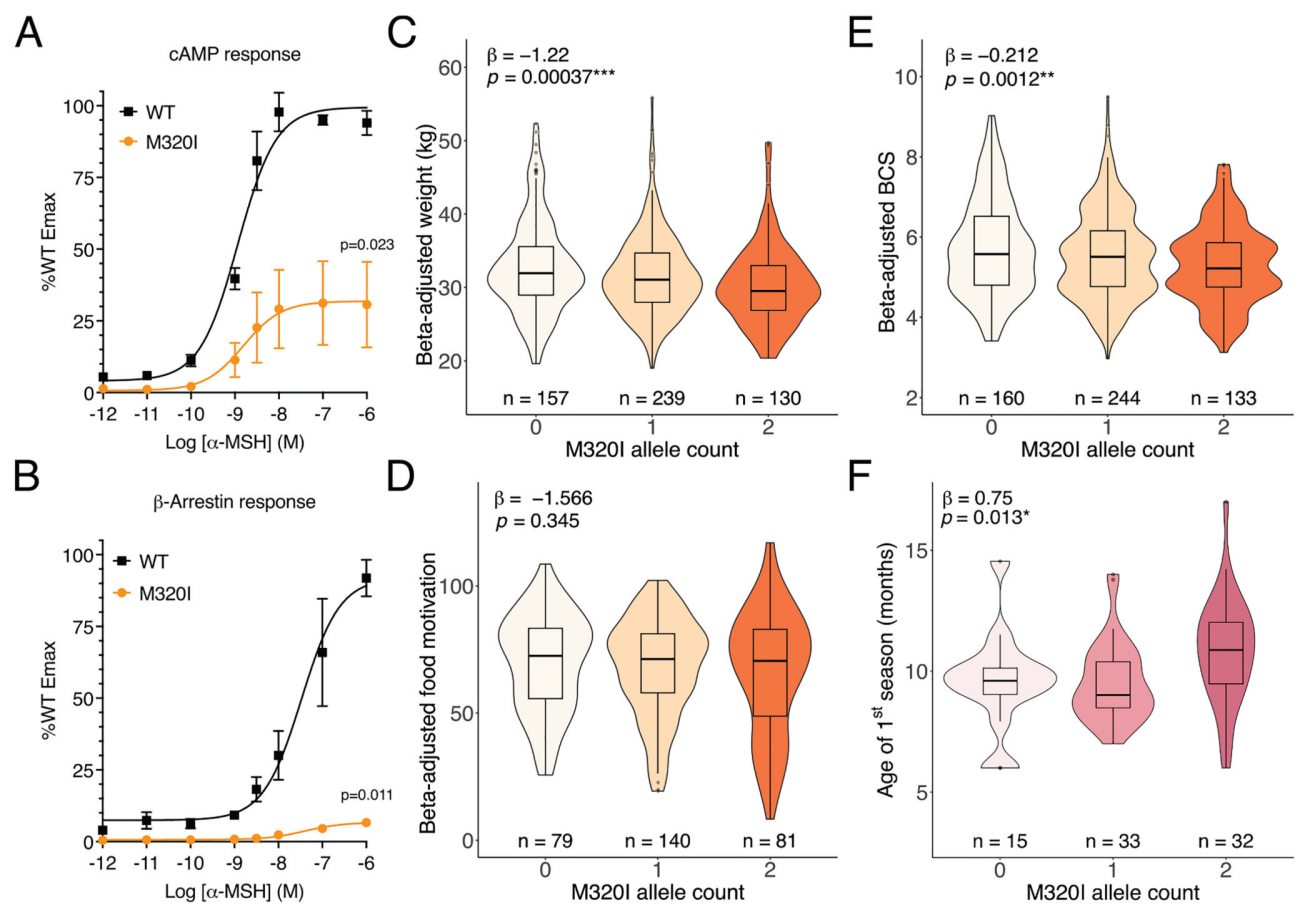
Loss of MC3R function is associated with delayed onset of puberty in dogs and reduced body condition score and weight. (A) cAMP generation and (B) β-arrestin recruitment measured *in vitro* for overexpressed *MC3R* construct, either WT or carrying the p.M320I mutation, upon stimulation with α-MSH. Error bars represent SEM. P-value reported for WT vs p.M320I Emax using unpaired two-tailed t-test. *N* = 3–4 biological replicates. (C—E) Partial regression violin plots for C) weight, D) food motivation and E) body condition score (BCS) from Labrador retrievers, per each allele dose of p.M320I. Test statistic and effect estimate obtained by ANOVA with allele effect modelled as additive. Beta-adjusted phenotypes calculated using residuals from linear regression coefficients for the respective phenotype (see methods for details). (F) Violin plots for 80 female, non-neutered Labrador retrievers and their age of first season. Overlayed boxplots show the median with 25th and 75th percentiles, and whiskers extend to 1.5 times the interquartile range.

We next examined a canine cohort to determine if dogs with impaired MC3R function exhibited similar anthropometric and pubertal phenotypes to humans and/or mice. Pet dog breeds have a more homogenous genetic background, and selective breeding means large effect mutations can become prevalent within breed populations which can be helpful for studying variant/phenotype associations. A cohort of 537 pet labrador retrievers had been phenotyped and genotyped at the *MC3R* locus [16]. The common (MAF = 0.48) missense mutation p.M320I was included on the genotyping panel and was characterised as having markedly impaired cAMP production in response to α-MSH (Figure 3A). The mutated receptor had almost absent β-arrestin recruitment, which is required for receptor internalisation [17] (Figure 3B).

The canine biobank has detailed phenotypes on body composition, including weight, body condition score (BCS) — a well validated measure that uses haptic and visual cues to assign a score for canine adiposity — and food motivation (FM) score, measured using an owner-reported questionnaire [18]. For each dog, the phenotype was adjusted using the residuals for age, sex and neuter status as identified using linear regression in the population. *MC3R* p.M320I carriers exhibit lower BCS and lower weight but their food motivation score was no different (Figure 3C−E).

To determine if MC3R-deficient dogs also exhibit delayed reproductive maturation, the age of first season (the observed signs of pro-oestrus/oestrus) in female dogs was investigated. Non-neutered, female labrador carriers of p.M320I exhibit delayed age of first season when carrying 2 copies of this LoF mutation (Figure 3F).

**Figure 4:**
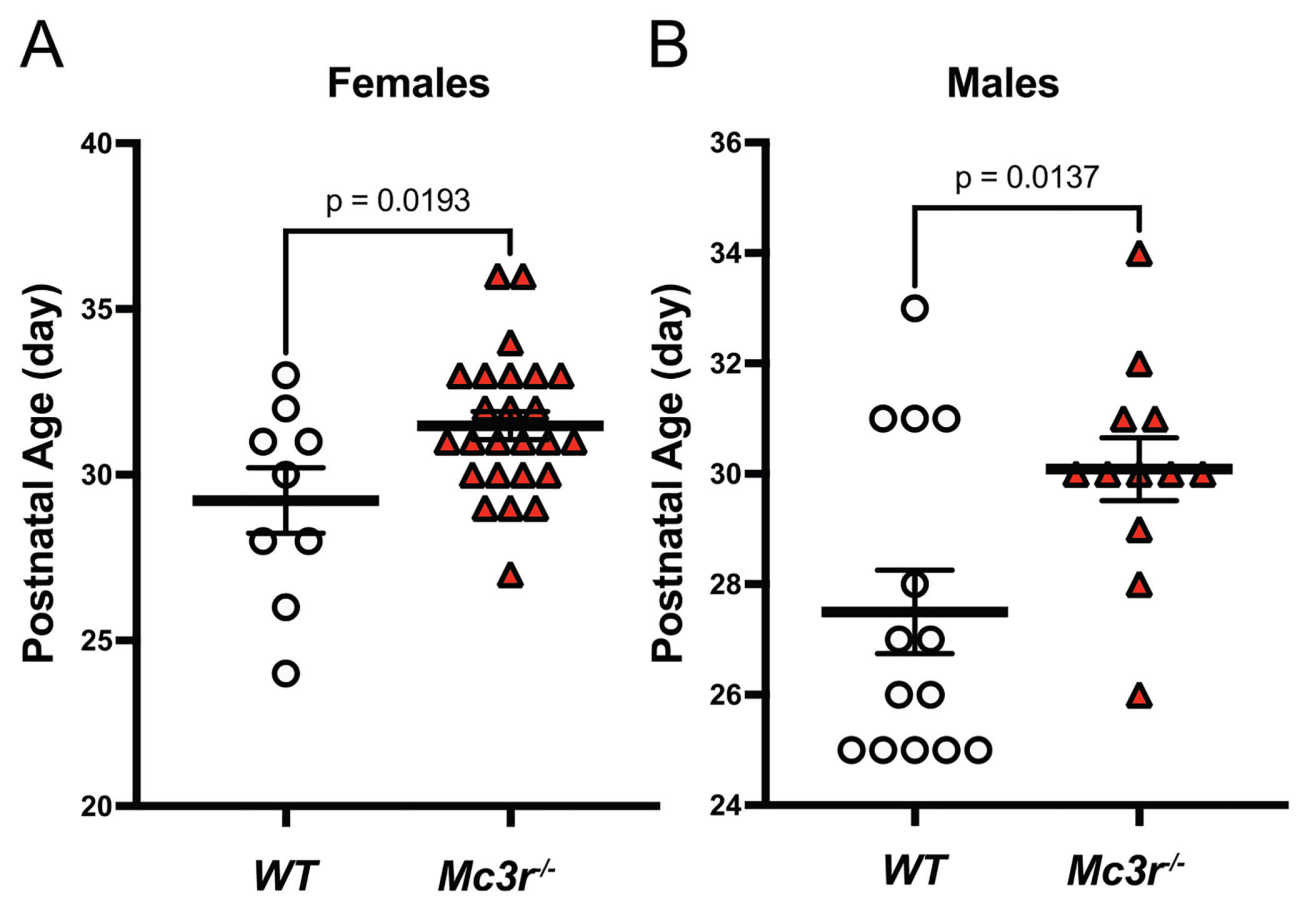
*Mc3r* deficient mice exhibit delayed pubertal onset (A) Age of vaginal opening in female mice and (B) age of preputial separation in male mice, in WT or *Mc3r*^-/-^ mice. Error bars represent mean and SEM. P-value calculated by unpaired, two-tailed t-test between genotypes.

In our previous work we reported that male mice lacking *Mc3r* had significantly delayed preputial separation, but did not find evidence for significant pubertal delay in females [9]. Considering that our earlier study may have been underpowered, we proceeded to phenotype larger, independent cohorts of male and female *Mc3r*^-/-^ mice and WT littermates. We confirmed the effect in males and established that age at vaginal opening is indeed significantly delayed in *Mc3r* deficient females (Figure 4).

## 3 Discussion

We have used a cross-species approach, involving humans, dogs and mice carrying LoF alleles of *MC3R* in an effort to provide clarity about the role of this receptor in the control of body weight, growth, and the timing of pubertal development.

In three independent populations from areas enriched in consanguinity (Pakistan, Bangladesh and Mexico) where data on height and weight had been recorded, we identified nine participants who were homozygous for functionally inactive *MC3R* variants. These variants were respectively enriched in people from these ethnic groups in gnomAD. Six of the nine did not have obesity and in the three individuals with obesity, the highest BMI was 31.7 kg/m^2^. Thus, it seems clear that the complete absence of MC3R function does not by itself result in a penetrant form of human obesity. This contrasts with homozygous/bi-allelic carriers of LoF mutations in other genes in the leptin-melanocortin signalling pathway -*LEP, LEPR, POMC, PCSK1* and *MC4R*-who almost inevitably develop severe obesity at an early age [19–23].

Some previous studies have reported that homozygosity for the common variant p.V44I/5′UTR haplotype is associated with increased BMI in childhood and adulthood [24–27]. However these studies were on small cohorts and other studies have not found general associations with p.V44I carriage and obesity [28–37].

Some studies have implicated rare heterozygous functional variants in *MC3R* as a contributor to early onset and/or common obesity [29,30,34,35,38–40]. A meta-analysis of these studies concluded that the carriage of such variants was associated with a 3-fold increased risk of having obesity [8]. It should however be pointed out that the studies contributing to this meta-analysis were characterised by small sample size and uncertainties about the prevalence of functional variants in the appropriately ethnically matched background population. In our previous study, we compared both BMI and fat mass of approximately 2400 unrelated heterozygous carriers of *MC3R* LoF variants to ~480k non-carriers from UK Biobank, and after matching for age, sex and other possible confounders we found no evidence of any impact of mutational status on either measure [9].

Of note, the *MC3R* locus has not been identified as a site for common genetic variation associating with obesity, waist-hip ratio or body fat percentage [41,42]. While it remains formally possible that some *MC3R* variants may contribute to increased BMI in certain populations, *MC3R* should not be considered a monogenic obesity gene and should not feature on diagnostic panels for genetic obesity.

The 9 homozygous carriers were not consistently shorter than the populations from which they came. This was surprising, given the clear association with heterozygous LoF carriage and reduced height in UK Biobank [9]. The heritability of height has been mapped to over 1000 loci (including *MC3R, p= 1E-131*) [43], and it is likely that the impact of MC3R is influenced by polygenic background of the different populations as well as their nutritional environment. The UK Biobank study participants are mostly of European ancestry and healthier and wealthier than the average UK population [44]. It is possible that nutritional and other environmental constraints on linear growth in childhood and adolescence are likely to be less pronounced in the UK Biobank than in the populations studied here. We speculate that the effects of *MC3R* (and indeed other) genotypes on final adult height may be more readily detectable in the absence of such nutritional constraints.

LoF mutations in *MC3R* have been robustly associated with a delay in the onset of puberty in males and females, and are enriched in cohorts of patients presenting with delayed puberty [45,46]. The single human homozygote for whom data has been available to date did not enter puberty until his third decade [9]. Unfortunately, data on the timing of puberty was not available in the three cohorts from which the homozygote mutation carriers were found. However, in the current work we have added to the body of data relating MC3R to human pubertal development by being able to directly assess pubertal phenotypes across time in a prospectively followed birth cohort, rather than relying on recalled data, as we did in our previous UK Biobank-based study. The power of this arm of our study was limited by small numbers of carriers but there was, nonetheless, some evidence that key male pubertal phenotypes were delayed in carriers of MC3R LoF mutations.

We report that three female homozygous carriers had children in their twenties, indicating lack of MC3R does not preclude fertility, consistent with the observation that the single male homozygous carrier of our previous report also fathered children [9].

The existence of a common functionally impaired *MC3R* allele in the Labrador dog population gave us an opportunity to examine the function of MC3R in another species. Dogs carrying the functionally impaired p.M320I variant had an allele dose-dependent decrease in body weight. Lower weight could be a consequence of reduced fat, lean, and/or bone mass. Mice lacking *Mc3r* have been described as either similar or lower weight to WT mice, attributed to the mice being smaller in length and having reduced lean mass [5,47–49]. Likewise, heterozygous humans are on average shorter with reduced lean mass [9]. Therefore, it is possible that these dogs also have lower length and reduced lean mass and are overall smaller, however we did not have the morphometric measurements required to confirm this.

The body condition score (BCS) is a validated, if only semi-quantitative, indicator of canine adiposity, with higher scores indicating greater adiposity [18]. Consistent with our findings in humans, there was no evidence for increased adiposity in dogs carrying the p.M320I variant, indeed their BCS was significantly lower.

While it is difficult for owners to provide information sufficiently accurate to time pubertal development of male dogs, in females “age at first season” (the age at which females start to exhibit sexually receptive behaviour) is more readily timed. Female dogs homozygous for p.M320I show a significantly delayed age of first season.

Our previous studies of sexual maturation in mice were of limited size. In our current work we studied an independent and substantially larger cohort of male and female *Mc3r* deficient mice and WT littermate controls and provide strong evidence for an effect of MC3R on age of reproductive development in both sexes.

Our study has limitations. Access to phenotypic data on the homozygous carriers from Pakistan, Bangladesh and Mexico City was restricted to information on height and weight. The canine study was also limited in phenotypic scope with no detailed measurements of body composition. In the ALSPAC study, where detailed phenotyping data was available, the modest number of *MC3R* LoF carriers limited our power to detect associations with pubertal phenotypes. Our functional assessment of MC3R variants was limited to assessment of cAMP responses. It is possible that the addition of other signalling endpoints, such as β-arrestin recruitment, may have increased our yield of LoF variants.

In summary, i) the complete congenital lack of MC3R does not result in any substantial increase in BMI in humans and therefore, in contrast to several other genes in the leptin melanocortin pathway, *MC3R* should not be included in diagnostic panels for “monogenic obesity”; ii) a functionally impaired missense variant in *MC3R* is common in labrador retriever dogs, where it is associated with lower body weight and does not predispose to obesity; and iii) MC3R status influences the timing of puberty across all three species studied.

## 4 Methods

### 4.1 Cohort descriptions for identification of homozygous carriers

To investigate the phenotypes of carriers homozygous for *MC3R* LoF mutations, three cohorts enriched for consanguinity were examined.

#### 4.1.1 BangladEsh Longitudinal Investigation of Emerging Vascular Events (BELIEVE)

A prospective cohort of ~73,883 participants recruited through a household survey between January 2016 and March 2020 [50]. Full details of the cohort are reported in [50]. Briefly, participants, who were aged 11–105 years at baseline (mean 39 years) and comprise ~60% women, were recruited from three different settings in Bangladesh: urban (Mirpur-Dhaka), rural (Matlab-Chandpur), and urban-slum (Bauniabadh-Dhaka). Participants were recruited irrespective of pre-existing disease. After the initial household visit, consenting participants were invited to attend a local study clinic to complete an individual-level baseline assessment, which included anthropometric measures obtained using standardised procedures and equipment. Height was measured using the ShorrBoard ICA Measuring Board to within 1 cm and weight was measured using the Tanita HD-661 scale to within 0.1 kg. The mean BMI of the urban population is 25.8 kg/m^2^, the urban slum population 23.4 kg/m^2^ and the rural population 22.6 kg/m^2^. WES was performed by the Regeneron Genetics Center, using the same methodology as described in [51] After QC, 71,942 participants remained for analysis. All coding, non-synonymous *MC3R* mutations with MAF<0.01 which were present in homozygosity were extracted for downstream analysis.

#### 4.1.2 Pakistan Genomics Resource (PGR)

A population biobank of multiple case—control cohorts for a wide range of diseases and phenotypes and is described in detail by [51]. The mean BMI of the population is 26.8 kg/m^2^. A total of 83,435 individuals of a range of ethnicities had either WES data (*N* = 75,819) or whole genome sequencing (WGS) data (*N* = 7,616). Sequencing and QC pipelines are described in [51]. *MC3R* coding, non-synonymous variants with a MAF<0.01 which were present in homozygosity were extracted.

#### 4.1.3 Mexico City prospective study (MCPS)

A prospective cohort of more than 150,000 adults aged 35 years or older, of predominantly admixed American ancestry, recruited between 1998 and 2004 from households within two urban districts of Mexico City. The cohort is fully described in [52]. Standing height was measured using a stadiometer, measured to the nearest millimetre. Weight was measured by calibrated electronic scales to the nearest 100g. The mean BMI of the entire cohort is 29.0 kg/m^2^ [52]. The WES sequencing was performed at the Regeneron Genetics Center and is fully described in Ziyatdinov et al. [53]. Whole exome data of 139,603 individuals was interrogated for presence of protein truncating variants of high confidence (HC PTV) based on LOFTEE prediction [54], and those variants present in homozygosity were extracted.

### 4.2 Canine cohort description and phenotyping

A biobank of 537 labrador retrievers from the GODogs research group was used in this study. Full ethics, recruitment, genotyping and phenotyping methods are described in [16]). *MC3R* genotypes were extracted from CanineHD 220k Array (Illumina) or Canine HD 712k Array (Axiom™), and the data aligned to CanFam3.1 using PLINK v2.00 [55].

Basic information on the dogs was collected, including breed, sex, neuter status and age. Dogs were excluded if they were under one year old so that puppy growth was not accounted for and if they were over 10 years old as older dogs have a higher frequency of disease that could affect appetite and weight. Other conditions and medication were excluded if they could affect appetite or weight including musculoskeletal injury/disease and gastrointestinal/metabolic disease.

#### 4.2.1 Body condition score (BCS)

BCS is a validated ordinal measure of adiposity in dogs using visual and haptic cues which overcome the diversity between breeds morphology that makes body mass index style measures invalid. It is a practical phenotype to score and used widely in veterinary clinics. In this study, all canine cohorts were measured by a veterinary professional using the Purina 9-point body condition system where <3 underweight, 4–5 = ideal, 6–7 = overweight and 8—9 = obese. This scale correlates highly with fat mass measured by Dual X-ray absorptiometry (DEXA) [18]. Dogs under BCS 4 were excluded from the study due to the likelihood of illness being determining unhealthy weight loss.

#### 4.2.2 Food motivation

Food motivation was determined from the owner reported DORA questionnaire, a previously validated tool for assessing canine eating behaviour [56]. Briefly, answers to questions on four and five option scales were converted to percentages of the maximum. Questions fell into three factor groups; food responsiveness and satiety, lack of selectivity and interest in food, and overall food motivation was calculated as follows to be expressed on a scale from 0 to 100:

$$\begin{array}{l} \text{Food}\;\text{motivation}=(\left(\text{sum}\;\text{of}\;\text{question}\;\text{scores}\right)\\ /(\text{sum}\;\text{of}\;\text{maximum}\;\text{question}\;\text{score}))\times 100 \end{array}$$

#### 4.2.3 Other quantitative phenotypes

Weight measurements were performed by veterinary professionals. Puberty timing in female dogs was determined by the age the dog started their first season, defined as the fertile period of a dog’s cycle that lasts approximately 16–18 days and is determined by observing clinical signs of oestrus.

### 4.3 Measuring cAMP production by human and canine *MC3R* variants

For human *MC3R* variants, cAMP signalling was assessed as previously described [46]. Briefly, HEK293 cells (ATCC) were plated into a 96-well plate and transiently transfected with WT or variant *MC3R* constructs for 48hrs. Cells were then treated with a final concentration range of 10^−14^ to 10^−5^ M NDP-MSH (Bachem) in phosphate buffered saline (PBS) for 2hrs. cAMP levels were measured using Eurofins DiscoverX Hithunter cAMP assay for Small Molecules as per the manufacturer’s protocol. Raw luminescence values were converted to cAMP concentration (μM) using a cAMP standard curve (~0–10 μM) and 3-parameter dose—response curves were fitted in R (v4.4.3) to calculate Emax and logEC50 values for WT *MC3R* and *MC3R* variants. All values were normalized to WT Emax (100%) and replotted for LoF classification. Variants were classified into the following categories: WT-like (75% WT < Emax ≤120% WT or 0.2 × WT ≤ EC50 < 5 × WT); PLoF (25% WT < Emax ≤75% WT or 5 × WT ≤ EC50 < 50 × WT); CLoF (Emax ≤25% WT or EC50 ≥ 50 × WT).

For the canine *MC3R* p.M320I variant, cAMP activity was measured live using the GloSensor assay (Promega). HEK293 cells were cultured in DMEM +10% FBS +1% Glutamax +1% Penicillin/Streptomycin. Cells were seeded into poly-D-lysine-coated 96-well plates at a density of 30,000 cells/well and incubated 24hrs. Cells were co-transfected with canine *MC3R* pcDNA3.1 (either WT, M320I, or empty vector control) (final conc.: 0.15 ng/μL) plus pGloSensor-20F plasmid (final conc.: 1 ng/μL) using Lipofectamine 2000 (Thermo Fisher) as per manufacturer’s protocol. 48hrs post-transfection, media was replaced with culture media with 2% GloSensor cAMP reagent stock solution. Cells were incubated in a Tecan Spark Multimode Microplate reader maintained at 37 °C 5% CO_2_ and baseline luminescence reading taken for 20 cycles of 30 s intervals. α-MSH ligand was added at a concentration range of 10^-7^ to 10^-12^ M, and 60 cycles of luminescence readings recorded.

Data was analysed in GraphPad Prism 8. Raw luminescence values for WT and M320I containing wells were normalised to the empty vector wells. AUC values were calculated for each dose and a 3-parameter log(agonist) vs response curve was fitted.

### 4.4 Measuring β-arrestin recruitment by canine MC3R

β-arrestin recruitment was assessed in HEK293 cells expressing MC3R using live NanoBit protein—protein interaction (PPI) system (Promega). Canine *MC3R* coding sequence (either WT or p.M320I) was cloned into a NanoBit PPI backbone to attach a C-terminal LgBIT tag, and β-arrestin construct was cloned into a SmBIT construct. HEK293 cells were plated as above and after 24hrs co-transfected with WT/variant-LgBIT (final conc.:0.5 ng/μL) plus β-arrestin-SmBIT (final conc.: 0.5 ng/μL) using lipofectamine 2000. Additional wells were co-transfected with WT receptor plus Halo-SmBIT to determine background random PPI signal. 48hrs post-transfection, cells were transferred to a plate reader as above. α-MSH ligand was added and luminescence recorded as above. The raw luminescence data was analysed using the same workflow as described for cAMP above.

### 4.5 Human homozygous carrier phenotype analysis

Following extraction of *MC3R* variants in the cohorts as described above, and *in vitro* identification of complete LoF variants, individuals were labelled as carrier of a CLoF variant if homozygous for that variant, or non-carrier if not homozygous for a CLoF variant.

Standing heights, weights and derived BMIs were extracted for all cohort participants. Manual inspection of individuals with extreme outlying heights and weights, which were very likely due to incorrectly recorded values, were removed.

For each cohort, each carrier’s age and sex were recorded. For BELIEVE, individuals were also stratified by recruitment setting. For PGR, individuals were stratified based on ethnicity. All non-carriers who were of the same sex, ethnic/demographic background, and in the same age bracket (defined as 18–24, 25–34, 35–44, etc), were extracted. A Z-score of this stratified population along with the carrier was then calculated for height, weight and BMI. This allowed all carriers to be plotted on the same scale relative to their background population. This analysis and plots were performed using R v4.4.3.

### 4.6 Canine phenotype analysis

All epidemiological statistics were performed in R (version 4.1.0). Data was organised using R package data.table v.1.14.8. Phenotypes and covariates were visualised using histograms and obvious outliers removed.

For each phenotype, a minimal linear model was calculated (without p.M320I genotyping status) by stepwise model selection using Akaike’s Information Criterion (AIC) by the base R function step(). This included age, sex and neuter status as covariates as well as interaction terms between covariates (except age of first season where covariates were not appropriate). The full linear model assessed was: lm(phenotype ~ sex + age + neuter + sex:age + sex:neuter + age:neuter) where colon represents an interaction term between two covariates.

Linear regression was performed on the corresponding phenotype’s minimal model where genotypes for p.M320I were coded 0, 1, 2 assuming an additive model of inheritance. Linear model effect sizes (β) for the genotype and covariates were obtained using the function summary().

To better visualise the genotype/phenotype relationship, the measured phenotypes were adjusted for the β for all covariates in the final linear model and displayed as partial regression plots. These were calculated using partial regression which calculates the residual phenotype as follows:

Residual phenotype value = individual phenotype value - (β covariate A * (covariate A - mean covariate A)) - (β covariate B * (covariate B — mean covariate B))

### 4.7 ALSPAC derivation of *MC3R* carrier status

Data from 10,094 out of a total 14,901 children of the Generation 1 (G1) of the ALSPAC cohort were used in this study [14,57,58]. Whole exome sequencing of 8,436 G1 children was performed as described in [13]). Coding variants at the *MC3R* locus were annotated using Variant Effect Predictor and Combined Annotated Dependent Depletion score (CADD). Coding variants which were either protein truncating variants, CADD ≥ 25 or variants previously known to have partial/complete LoF were included and were carried by 51 children in the WES dataset [9,29,59]. In addition, a single carrier of p.A214P, a variant not captured in the WES but validated by Sanger sequencing, was included as a carrier [9]. An additional 6 carriers of partial LoF variant p.R220S were captured in the HRC imputed genotyping panel of 9,115 G1 children (Illumina 550 quad + Haplotype Reference consortium panel). This gave a total of 58 carriers and 10,036 non-carriers to be included in downstream analyses.

### 4.8 ALSPAC puberty phenotype analysis

Nine puberty phenotypes were derived from longitudinal data as described in [15]). Of the 10,094 individuals with genotype data, 7,656 had puberty data (including 42 out of the 58 *MC3R* LoF carriers). All phenotypes were normally distributed with the exception of age at axillary hair. For each pubertal phenotype for each sex, a linear model was run with each puberty phenotype as the dependent variable and *MC3R* LoF carrier status set as a predictor. No additional covariates were included. For phenotypes relevant to both sexes, values were standardised within sex then combined to enable sex-combined analyses.

### 4.9 ALSPAC anthropometric phenotype analysis

Data on weight, height and BMI were available from birth to average age 24 (a total of 20 timepoints). Fat mass and lean mass were available at ages 9, 11, 14, 15, 18 and 24 years and were obtained from DEXA scans performed using a Lunar Prodigy scanner (Lunar Radiation Corp). For ages 22 years and onwards, study data were collected and managed using REDCap electronic data capture tools hosted at the University of Bristol [60]. Supplementary Table 5 provides the number of participants for whom data was available at each timepoint. Linear-mixed effects models with restricted cubic splines for age were used to model trajectories of anthropometric traits from childhood to early adulthood. A model with 2 knots was used and the choice of knots location was based on the observed distribution of the anthropometric traits. For BMI, knots were placed at ages 5 and 10 years, for height knots were placed at ages 9 and 20 years, and for weight at ages 5 and 15 years. For lean and fat mass, knots were placed at ages 15 and 18 years. Models were adjusted for sex, and an interaction term between carrier status and age was included.

### 4.10 Mouse puberty phenotyping

C57BL/6J (B6) *Mc3rTB/TB* line (*Mc3rtm1Butl/J*), originally obtained from Jax #017866 and previously described [47], carries a loxP-flanked transcriptional STOP cassette (loxP-STOP-loxP) inserted into the 5′ untranslated region, which inhibits *Mc3r* expression. Cohorts of *Mc3r*^−/−^ mice and WT littermates were obtained from het × het breeding pairs and kept under controlled light (12hr light:dark cycle (06:00:18:00), temperature (22 ± 1 °C) and humidity conditions (45−65%) in individually ventilated cages with ad libitum access to food (RM3(E) Expanded Chow (Special Diets Services)) and water. Beginning at 3 weeks of age, puberty onset in WT and *Mc3r*^−/−^ mice was assessed by daily monitoring of preputial separation in males and vaginal opening in females.

### 4.11 Statistics

For human *MC3R* variant cAMP measurements, statistical difference from WT for Emax and logEC50 non-normalised values was determined using ANOVA for all variants, with variant, plate number, and day of assay as covariates in the model, followed by post hoc Dunnett test to determine statistical significance for each variant (all performed using Rv4.4.3). For p.A149T, p.L262V and p.I298S, these variants were characterised separately and were analysed Graphpad Prism v10.4.2. Paired two-tailed t-tests were performed for Emax and logEC50 for each of these variants vs WT.

For canine *MC3R* variant cAMP and β-arrestin measurements, raw pEC50 and Emax values from each replicate were collated, then WT vs p.M320I compared using unpaired two-tailed t-tests.

For canine phenotype linear models, statistical association of geno-types with each phenotype was assessed using the ANOVA test with R packages: base R and car.

For ALSPAC puberty phenotypes, p-values from two-tailed t-test on the coefficient of carrier status from the linear models are reported. For age at axillary hair which had a non-normal distribution, an additional Spearman’s rank test was performed with little difference in p-value compared to the linear model.

For ALSPAC anthropometric trajectories, the interaction between *MC3R* carrier status and age was tested using a Wald test.

P-values reported for differences between WT and *Mc3r* deficient mice were calculated by unpaired, two-tailed t-test between genotypes.

### 4.12 Study approval

Ethical approval for the study was obtained from the ALSPAC Ethics and Law Committee and the Local Research Ethics Committees, details of which can be found at http://www.bristol.ac.uk/alspac/researchers/research-ethics/. Informed consent for the use of all data collected was obtained from participants following the recommendations of the ALSPAC Ethics and Law Committee at the time. The completion of a questionnaire, either on paper or online, was considered to be written consent from participants to use their data for research purposes. For the majority of tests undertaken during face-to-face visits, verbal consent was obtained from participants (both parents and children as appropriate) prior to the start of any data collection. However, some tests required the completion of a written consent form. Study participation is voluntary and during all data collection sweeps, information was provided on the intended use of data. Biological samples are collected in accordance with the Human Tissue Act (2004). Specific Research Ethics Committee approval is sought for the consenting process at each collection sweep. Written consent, including permission for future use, is obtained from adult participants or from the parents of children as appropriate. Ethical approval for future use is covered by ALSPAC’s Research Tissue Bank approval. All historical consents to hold biological samples have been reviewed as part of the Tissue Bank approval process. Participants can contact the study team at any time to retrospectively withdraw consent for use of their samples or data.

The BELIEVE study has received approvals from the relevant institutional review boards of the Bangladesh Medical Research Council, the National Heart Foundation Hospital and Research Institute, icddr,b and Bangabandhu Sheikh Mujib Medical University (BMRC/NREC/2013—2016/390; BMRC/NREC/2016–2019/243; BSMMU/2019/1184; BSMMU/2019/1185; PR-18051; HBREC.2019.09). Written informed consent has been obtained from each participant (or by a parent or guardian for participants under the age of 18), including for future use of data and stored samples and invitation to further research studies. The MCPS study was approved by scientific and ethics committees within the Mexican National Council of Science and Technology (0595 P-M), the Mexican Ministry of Health and the Central Oxford Research Ethics Committee (C99.260), and the Medical Ethics Committee of the National Autonomous University of Mexico (FMED/CEI/MHU/001/2020). Study participants gave signed consent in keeping with accepted ethical practices at the time for observational cohort studies.

The PGR study was approved by institutional review board (IRB) at the Center for Non-Communicable Diseases (IRB: 00007048, IORG0005843 and FWAS00014490) approved the study. This study has also been approved by the National Bioethics Committee for Research Pakistan (reference number NBC-756). All participants gave written informed consent.

All mouse studies were performed in accordance with UK Home Office Legislation regulated under the Animals (Scientific Procedures) Act 1986 Amendment Regulations 2012 following ethical review by the University of Cambridge Animal Welfare and Ethical Review Body (AWERB).

For canine studies, the research was approved by the Ethical Review Committee of the Department of Veterinary Medicine, University of Cambridge (CR73 and CR125), with sample collection at other centres also approved by local ethical review committees: Animal Health Trust Research Ethics Committee, MIT Animal Care Protocol Lindblad-Toh 0913-073-16, Veterinary Ethical Review Committee of the University of Edinburgh (VERC 11/12), University of Liverpool Research Ethics Committee RETH000353, Swedish Animal Ethical Committee (C138/12, C62/10, and C2/12), and the Swedish Animal Welfare Agency (no. 31–1711/10). All dog owners gave full written consent to participate in the research.

## Supplementary Material

Supplementary data to this article can be found online at https://doi.org/10.1016/j.molmet.2025.102301.

Supplementary Material

## Figures and Tables

**Table 1:** List of homozygous mutations found in PGR, BELIEVE and MCPS cohorts.

Cohort	Variant ID (GRCh38)	Rsid	Amino acid change	CADD score	Cohort MAF	N Hom (ALT)
**PGR**	20:56249030:G:A	rs143370838	p.V63M	24.9	0.00043	2
**PGR**	20:56249093:G:A	rs754750284	p.D84N	25.6	0.00017	1
**PGR**	20:56249115:A:G	rs201051836	p.N91S	25.6	0.00013	1
**PGR**	20:56249134:G:A	rs199684791	p.M97I	21.3	0.00400	12
**PGR**	20:56249273:G:C	rs775292393	p.V144L	21.8	0.00007	1
**MCPS**	20:56249272:C:G	rs148382606	p.Y143*	35	0.00384	4
**BELIEVE**	20:56249134:G:A	rs199684791	p.M97I	21.3	0.00446	4
**BELIEVE**	20:56249030:G:A	rs143370838	p.V63M	24.9	0.00074	2

Variant ID reported as chr:loc:ref:alt. Amino acid change based on NCBI NP_063941.3 sequence. CADD=Combined Annotation Dependent Depletion (bioinformatic prediction of deleteriousness, with higher score indicating greater deleteriousness). MAF = minor (alternate) allele frequency.

## Data Availability

Data will be made available on request.
