# Supplementary material for: Cross-species studies implicate the melanocortin 3 receptor more strongly in the control of pubertal development than energy balance
Source: Mol Metab. Author manuscript; Available in PMC 2026 Jan 23. (PMC12808844; doi:10.1016/j.molmet.2025.102301)
Supplement: Supplementary Material [file EMS211997-supplement-Supplementary_Material.zip › 1-s2.0-S221287782500208X-mmc2.pdf]

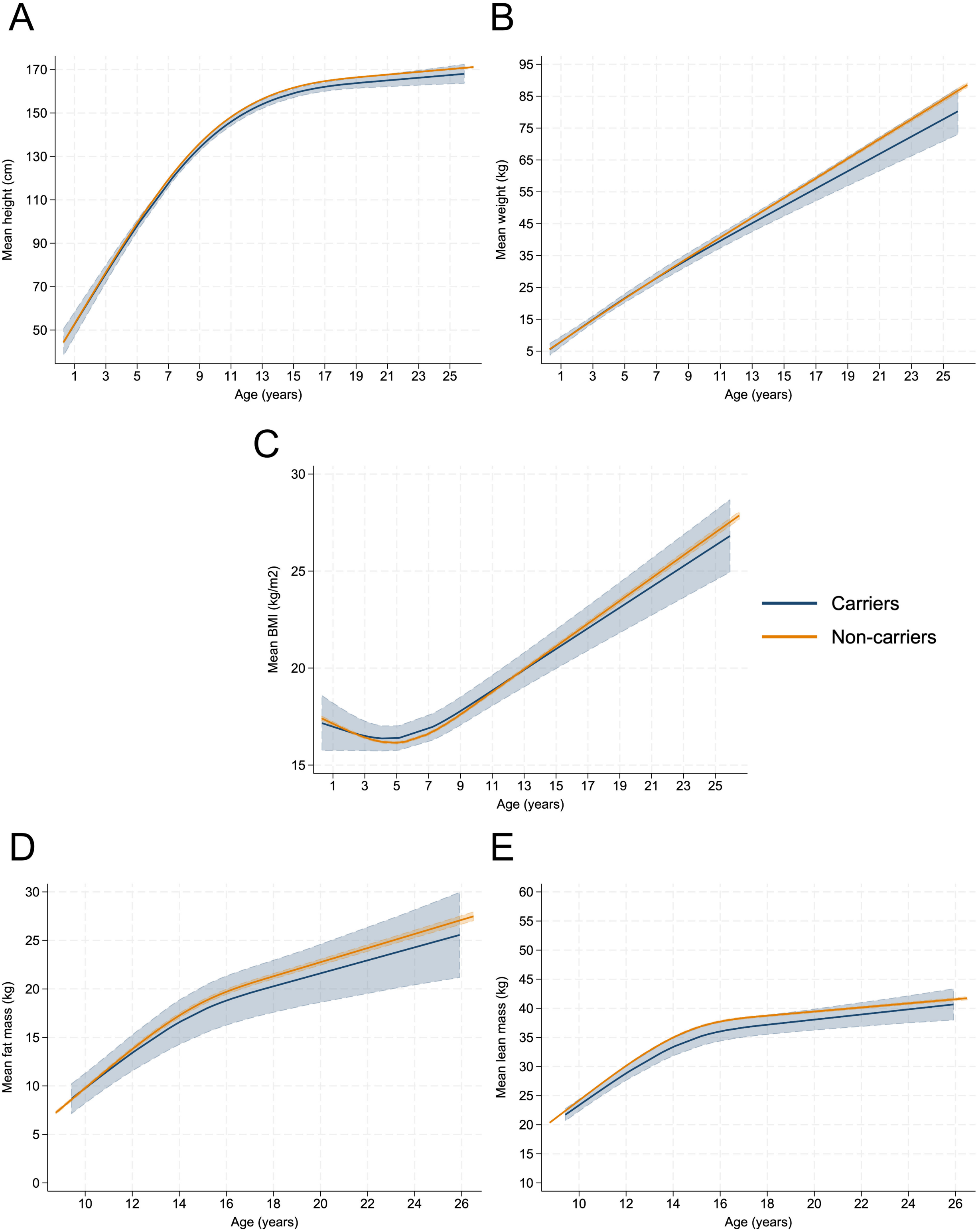


**Supplementary Figure 1: Trajectories of anthropometric measures in ALSPAC individuals carrying loss of function *MC3R* variants vs non-carriers.** (A-E) Models of (A) height, (B) weight, (C) BMI, (D) fat mass and (E) lean mass plotted for carriers and non-carriers of *MC3R* LoF variants. Shaded blue area represents 95% confidence estimates for carrier trajectories. Numbers of individuals used in each trajectory can be found in Supplementary Table 5.
